# Strengthening the System Supporting Perinatal People with Substance Use Disorder in the Midwest Using Group Model Building

**DOI:** 10.1007/s10995-023-03751-z

**Published:** 2023-07-21

**Authors:** Jessica Simon, Isabella Guynn, Meagan Thompson, Sarah Hambright, Cresta Jones, Kristen Hassmiller Lich

**Affiliations:** 1 Association of Maternal and Child Health Programs, 1825 K Street NW, Suite 250, Washington, DC 20006 USA; 2 Department of Maternal and Child Health, National MCH Workforce Development Center, University of North Carolina at Chapel Hill, 412 Rosenau Hall, Chapel Hill, NC 27599 USA; 3 Department of Health Policy and Management, Gillings School of Global Public Health, University of North Carolina at Chapel Hill, 1105E McGavaran-Greenberg Hall, Chapel Hill, NC 27599 USA; 4 Susan S. Morrison School of Nursing, University of St. Thomas, 2115 Summit Ave, St. Paul, MN 55105 USA; 5 Department of OBGYN, Maternal Substance Use Disorder Clinic, University of Iowa, 200 Hawkins Dr, Iowa City, IA 52242 USA; 6 Department of Obstetrics, Gynecology and Women’s Health, University of Minnesota Medical School, 606 24th Ave S, Suite 401, Minneapolis, MN 55455 USA

**Keywords:** Perinatal substance use, Causal loop, Group model building, Strategic planning, Mental models

## Abstract

**Introduction:**

Providing comprehensive, evidence-based care to perinatal people with substance use disorders (SUD) requires multi-stakeholder collaboration and alignment. The National Maternal and Child Health Workforce Development Center facilitated a system-strengthening process with the Midwest substance use in pregnancy (SUPper) club, a regional collaborative of health care providers, state public health agencies, and community-rooted organizations.

**Methods:**

Facilitators led a 2 day group model building (GMB) workshop with 20 participants and two semi-structured interviews. Workshop participants were invited to complete an evaluation.

**Results:**

Two primary trends were identified as priorities for change: (1) Birthing people’s perception/experience of stigma and (2) The Midwest SUPper Club’s reach and influence. Three causal loop diagrams (CLDs) were created to capture the interconnected dynamics of the Midwest perinatal SUD system: (1) the influence of stigma on maternal and infant health outcomes, (2) the role of clinic, organizational, and state policies, and (3) the impact of workforce education and evidence-based practices on care. From the CLDs, four priorities for action emerged: (1) align and promote shared mental models across stakeholders, (2) expand education and training opportunities for the perinatal SUD workforce, (3) strengthen systems infrastructure to support care navigation for patients and providers, and (4) collaboratively identify evidence-based practices that meet regional needs. All evaluation respondents reported that the workshop supported the development of a shared mental model.

**Discussion:**

The GMB process strengthened collaboration and advanced strategic planning for the SUPper Club. GMB can be further utilized among diverse stakeholders across MCH systems to create shared mental models and accelerate collaborative planning efforts.

**Supplementary Information:**

The online version contains supplementary material available at 10.1007/s10995-023-03751-z.

## Significance

*What is already known on this subject?* Perinatal SUD is a complex public health challenge, particularly in the Midwestern United States, where rurality and workforce shortages are common. Prevailing approaches often use siloed strategies rather than integrating the complex, interrelated dynamics and perspectives of relevant stakeholders.

*
What does this study add?* This is the first known use of GMB with perinatal SUD stakeholders, which we show to be a valuable tool for furthering stakeholder collaboration and action planning. State and local MCH programs should consider using these GMB and systems-thinking approaches, as demonstrated, in their own system-strengthening efforts.

**Supplementary Information:**

The online version contains supplementary material available at 10.1007/s10995-023-03751-z.

## Introduction

Providing effective and holistic substance use disorder (SUD) prevention, treatment, and recovery services remains a major public health challenge in the United States and is of significant concern for maternal and child health populations. Perinatal SUD too often coincides with punitive measures and criminalization, rather than the evidence-based treatment proven to effectively support recovery, such as medication for opioid use disorder (MOUD) and peer support (Saia et al., 2016). Even when treatment is available, pregnant and parenting individuals experience lower rates of treatment retention due to time constraints, fear of child removal from custody, and stigma among healthcare providers (NIDA, 2020; Trainor, 2022).

Rural communities face unique challenges in addressing substance use disorders. Compared to urban areas, rural communities have higher overdose death rates (Centers for Disease Control & Prevention, 2017) and numerous barriers to providing comprehensive treatment. These barriers include a lack of transportation, limited employment opportunities, unaffordable housing, inadequate health insurance, and limited access to recovery support groups (Clark et al., 2021; Palombi et al., 2019). Additionally, the limited medical infrastructure in rural areas coupled with the stigmatization of substance use disorders makes MOUD difficult to access (Dombrowski et al., 2016). These challenges are particularly prevalent in the Midwest, where much of the region is rural, and overdose deaths rates continue to increase despite declines in parts of the Northeast (Ahmad et al., 2022). While the opioid epidemic has drawn widespread attention and national resources, methamphetamine use has largely been overlooked as a public health concern and is growing in prevalence in the Midwest (Courtney & Ray, 2014; Hedegaard et al., 2019). Unlike most other non-prescribed substances, methamphetamine use appears equally as prevalent in women as in men (Courtney & Ray, 2014).

The complexities of substance use among both perinatal populations and Midwestern populations demand an innovative approach to better understand interactions between perinatal people who use substances, public health systems, community-rooted services, and clinical providers. Applying a systems perspective to perinatal substance use allows for diverse stakeholders to collectively produce insights on the underlying dynamics driving outcomes, with the goal of informing strategic planning and policy efforts (Hovmand, 2014; Naumann et al., 2021; Sterman, 2000).

The National Maternal and Child Health (MCH) Workforce Development Center, a technical assistance center supported by the federal Maternal and Child Health Bureau (MCHB) since 2013, is uniquely positioned to accelerate strategic planning utilizing systems thinking tools (Coffey et al., 2022). The Workforce Development Center has over nine years of experience responding to emerging needs through tailored trainings and workshops for Title V programs and their partners in three core areas: systems integration, change management/adaptive leadership, and evidence-based decision-making.

In this paper, we highlight findings from a system-strengthening process facilitated by the National MCH Workforce Development Center with members and potential partners of the Midwest^1^ Substance Use in Pregnancy Club (Midwest SUPper Club). The Midwest SUPper Club is a multi-disciplinary, grassroots regional coalition of perinatal and substance use providers, state public health agencies, and community-rooted organizations. The coalition formed in 2020 to address the need for Midwest-specific interventions, knowledge sharing, and collaboration (Zaman et al., 2023).

## Methods

Qualitative group model building (GMB) and key informant interviews were used to identify opportunities for the Midwest SUPper Club to effectively leverage their collective expertise to advance outcomes for people with perinatal SUD. GMB is a community-based applied systems thinking methodology to elicit systems structures, stakeholder mental models,^2^ and insights about how to meaningfully improve system behavior (Hovmand, 2014; Muttalib et al., 2021; Sterman, 2000). GMB differs from other stakeholder engagement methods in its use of facilitated, scripted activities to apply evidence-based components of systems thinking, as well as its strong emphasis on exploring potential positive and negative short- and longer-term impacts of policy and intervention options (Hovmand et al., 2012). GMB activity scripts are utilized to support better implementation of the GMB method and ensure fidelity to the process (Hovmand et al., 2012). In addition to the system-level insights produced through GMB, the process itself has the capacity to strengthen stakeholder collaboration by generating buy-in and providing a shared language for understanding the system. The MCH Workforce Development Center received Institutional Review Board approval for workforce development activities through the host university (IRB #14-0768).

### Group Model Building

Midwest SUPper Club members and potential partners were invited to take part in a 2 day (10 h) virtual GMB workshop. Recruitment efforts were pragmatic, relying on SUPper Club leadership^3^ to extend personal invitations to existing partners and, when prompted by the workshop facilitators, to seek out additional attendees from state public health agencies, community-based organizations, and individuals with lived experience. A small stipend was offered for people with lived experience to attend. Recruitment intentionally centered around including perspectives from diverse sectors in the broad perinatal SUD treatment system in the Midwest. Twenty participants from four states (Iowa, Michigan Minnesota, and Wisconsin) participated in at least one day of the workshop, which reflects the Midwestern states in which the SUPper Club has had the highest level of engagement. Collectively, the organizations and individuals participating in the workshop brought rich insight into unique challenges of many sub-populations seeking out perinatal SUD care in the Midwest, including geographic, education-level, cultural, and racial/ethnic sub-populations. Roughly half of the participants were clinicians from various hospital/health-care systems (N = 10), with the other half representing public health agencies or organizations (N = 9) or lived experience of perinatal substance use disorder (N = 1). Due to scheduling conflicts, 19 participants attended Day 1 and 10 participants (9 returning and 1 new) attended Day 2.

Three facilitators trained in GMB planned and led the workshop following a series of scripted GMB activities described in Table 1 that were modified for the virtual context (Andersen & Richardson, 1997; Chin et al., 2022; Hovmand et al., 2012, 2015). The workshop was facilitated via Zoom, using both Google Slides and Vensim (a causal mapping and simulation software) to support activities. With permission, main session discussions were recorded for internal note-taking purposes and to capture meaningful quotes from participants. Following Day 1, in the three-day interval between workshops, facilitators reviewed the recording and all annotated Google Slides in order to develop final version(s) of the causal loop diagrams (CLDs). On Day 2, facilitators introduced the updated CLDs and initiated small group discussions to capture missing elements and insights. Finally, leverage points and corresponding priorities for action were elicited from the CLDs by participants.

**Table 1:** Description of group model building workshop activities

GMB activity*	Inputs, activity description, and products
Hopes and fears	The workshop sequence began with “Hopes and Fears” to unearth participants expectations for the workshop and their work together.
Behavior-over-time graphs (BOTGs)	The systems-strengthening process continued by defining the problem through Behavior-Over-Time Graphs (also referred to as “Graphs Over Time”). This activity asked all participants to graph values over time for a variable (or set of variables) that they thought were most important to address, and to share the story they captured in their graph(s) with the group to illuminate key factors driving outcomes within the system (Calancie et al., 2018; Sterman, 2000). By the end of the activity, two primary trends were selected and agreed upon to focus on influencing as a group.
Connection circles	Participants reflected on annotated graphs from the previous activity and identified 6–8 relevant variables shaping the primary trends over time. These variables were placed around a circle and arrows were drawn between variables when there was a causal linkage between them using a variation of the “Connection Circles” script. Facilitators prompted for stakeholder perspectives that may not have been fully represented by participants.
Causal loop diagramming	During a break in the workshop, facilitators replicated and integrated the connection circles from three small groups in Vensim to create one initial causal loop diagram (CLD). Facilitators then led participants through reviewing and iterating the diagram to incorporate feedback given one at a time following a normative group process.
Model review and iteration	After the first day of the workshop, the facilitation team carefully reviewed the CLDs to ensure all major variables, pathways, and feedback loops were captured. Because the diagram became overwhelmingly large, facilitators separated the diagram into three CLDs to represent the major themes that had emerged. The updated CLDs, along with any clarifying questions, were discussed and further revised on day two of the workshop using the “Model Review” script.
Leverage points for action	The workshop concluded with an adapted version of the “Action Ideas” script. Participants reflected on the three CLDs and levels of leverage to brainstorm and reach consensus on four high-priority leverage points, then identified and prioritized specific action ideas for each.

*GMB activities were based on the original scripts from Scriptapedia (Hovmand, et al., 2015)

### Semi-Structured Interviews

Pairs of interviewers trained in GMB conducted semi-structured interviews with two subject matter experts from Iowa and Wisconsin whose schedules did not allow for them to attend the workshop. Both individuals were health care providers with a combined 14 years of experience directly serving perinatal people with SUD, and had been identified by SUPper Club leadership as having an important perspective to share. Each interview lasted approximately 60 min and was conducted virtually via Zoom. Interviewers first presented a summary of workshop deliverables, specifically focusing on the CLDs, and then asked for feedback to corroborate findings from the GMB workshop and continue refining the CLDs.

### Workshop Evaluation

Following the workshop, participants were asked to complete a 22-question evaluation via Qualtrics. This evaluation was created by a member of the evaluation team at the MCH Workforce Development Center. The short survey, which was distributed to participants via an emailed survey link, included both multiple choice and open-ended questions on the workshop’s impact on system thinking skills at an individual and team level.

## Results

### Behavior-Over-Time Graphs

In two groups, participants developed a total of 20 behavior-over-time graphs (BOTG) during the workshop, capturing meaningful trends that the SUPper Club could focus change efforts on. The groups reached consensus through iterative discussion on two primary trends that capture the club’s role in strengthening the perinatal SUD system (presented in the Supplementary Material) : (1) Birthing peoples’ perception/experience of stigma from the treatment community, which was viewed as “high stigma” in the past and present, with a hope to decrease to “low or no stigma” in the future; and (2) The Midwest SUPper Club’s reach and influence, which has demonstrated deliberate growth since its inception in 2020, with a hope to avoid stagnation and continue to expand engagement in the future.

### Causal Loop Diagrams

The three CLDs that emerged to capture separate but interrelated dynamics include: (1) the influence of stigma on maternal and infant health outcomes, (2) the role of clinic, organizational, and state policies, and (3) the impact of workforce education and evidence-based practices on care. See Tables 2, 3, 4 for detailed descriptions of the feedback loops identified in each model (including guidance on how to read and interpret CLDs) and the Supplementary Material for a list of endogenous and exogenous variables elicited.

**Table 2:** Feedback loops in the stigma and MCH outcomes model

Name of feedback loop	Feedback loop structure	Explanation of feedback loop	Participant quotations
Example		An **“S”** on the arrow indicates that the two connected variables move in the **same direction** (i.e., if the first variable increases, it triggers an increase in the second variable; if the first variable decreases, the second variable decreases). An **“O”** on the arrow indicates that the two variables move in **opposite directions** (i.e., if the first variables increases, the second variable decreases; if the first variable decreases, the second variable increases).	
		This example shows that an individual/community response to increasing stress levels is to support development of coping strategies to deal with the stress. This **balancing feedback loop** (“B”) is striving to “control” stress and keep it at a tolerable level, and assumes that increased use of coping strategies will effectively reduce stress. Coping strategies should thus be enhanced in individuals and strengthened over time to retain this defense against stress.	
R1: Perpetuation of structural and social stigma		High levels of institutional and systems-level stigmatization of perinatal substance use drives individual stigma perpetuated by people working in the prevention, treatment, and recovery community. This individual-level stigma further fuels/creates/increases structural stigma, in a **reinforcing feedback loop** (“R”) explaining increasing cycles of growth (or decline, if either is pushed to decrease) in inter-connected social and structural stigma.	“*When our health system is unwilling to invest in perinatal addiction treatment, it does send a message to those of us in clinical care that this is not something to be prioritized during pregnancy, and further reinforces stigma.”—Obstetric Care Physician*
R2: Provider stigma limits patient centered care		Social stigma limits the provider’s desire to provide patient-centered care by consciously or subconsciously acting on the belief that substance use is a choice and not a medical condition. Alternatively, a strong desire to provide patient-centered care can challenge and reduce the prevalence of social stigma.	*“When they come in to deliver, for instance, the group that's on may or may not have, the same level of education, or examined their own biases, or done any of the things that we need to do to really provide quality care to these folks.”—Hospital Nursing Director of Women’s Health*
B1: Poor infant health outcomes creates urgency to improve care		A lack of high-quality, patient-centered, equitable, evidence-based, and trauma-informed care negatively impacts infant health outcomes, including through increased exposure to adverse childhood experiences (ACEs). Poor infant health outcomes increase the provider/service communities focus on reducing prenatal substance exposure, including through the delivery of more equitable and evidence-based care. While this solves the problem of improving care in the short-term, it raises questions about sustaining investment and engagement in improving perinatal substance use care in the long-term.	*“We focus significantly on reducing prenatal substance exposure, which is important, but we have limited guidance on how to provide evidence-based support for pregnant people and infants exposed to non-prescribed substances (i.e., a harm-reduction based model of care) and I worry that this may negatively impact patients and families in the long term.”—Maternal Fetal Medicine and Addiction Medicine Physician*
R3: Interdependence of maternal and infant health outcomes		When birthing people experience positive health outcomes, including SUD and pregnancy/postpartum outcomes, their infants are more likely to experience positive outcomes, such as lower rates of preterm birth and a decreased likelihood of neonatal abstinence syndrome, among others (Patrick et al., 2017). Similarly, birthing people may experience more positive mental health outcomes as a result of a healthy newborn infant.	*“When you treat the pregnant person, you are also investing in their health across their lifespan and potentially the lifespan of the infant. You cannot focus only on treatment of one without thinking of the benefits to the other. If you only treat the neonate, you forsake the health of the mother who wants to provide skin-to-skin, the mother who wants to bond, the mother who wants to breastfeed. The mother who desperately wants to get better.”—OBGYN Provider*
R4: Self-stigma drives maternal health outcomes		A birthing person’s self-stigmatization and negative beliefs about their SUD and capacity for recovery can discourage engagement in perinatal and SUD care, which decreases the likelihood of positive health outcomes. Negative health outcomes can perpetuate self-stigma by reinforcing the negative beliefs a person holds about themselves and their SUD.	*“Patients often already carry so much guilt and self-blame due to their substance use and substance use disorders. And when they experience a negative health outcome, it may reinforce their beliefs that they are not deserving of good things in their lives.”—OBGYN Provider*
R5: Self-stigma drives infant health outcomes		Similarly to loop **R4**, a high level of self-stigma in the absence of perinatal and SUD care negatively influences infant health outcomes. Poor infant health outcomes reinforce self-stigma as birthing people may feel selfblame without adequate support and treatment opportunities.	*“Self-stigma, shame, fear and guilt all contribute to pregnant people with SUD not engaging in prenatal care. These thoughts, emotions and feelings impact the birthing person’s health as well as infant health outcomes. It is crucial to acknowledge and educate the care team, and remind the patient, that a SUD is a disease and can be treated. It is recommended to have SUD screening for every OB patient in the prenatal setting to allow for safe, therapeutic and trauma-informed conversation about SUD during pregnancy. Reminding the patient that the care team is supportive, that there is treatment for SUD, and remaining non-judgmental can lead to patient centered goals for reduced use or abstinence to improve maternal and infant outcomes.”—OBGYN Social Worker*
R6: Engagement in care improves health outcomes/encourages continued engagement		When birthing people are highly engaged in perinatal and/or SUD care and they experience positive health outcomes, they are more likely to remain engaged in future care.	*“If there’s a feeling of stigma from past experiences, patients may not be seen for care and that absence of care may be associated with undesirable outcomes.”—OGBYN/Addiction Medicine Physician*
R7: Family separation harms birthing peoples’ health outcomes		Family separation, whether from parental incarceration or child protective service intervention, is detrimental to maternal behavioral health and has been linked to increased substance use, further reducing the likelihood of family reunification (Harp & Oser, 2018).	*“I’ve worked with parents who really want to get to where they needed to be, but they just didn’t have enough time to do it. People are going to relapse and need more support… and now all the sudden the county is pushing permanency and it sets the parents up for failure under that kind of pressure”—State Department of Health staff member*
R8: Family separation harms infant health outcomes		Family separation harms infant health immediately and over the life-course through its negative impact on parent child attachment, breastfeeding, childhood behavioural health, and increased exposure to ACEs (Lander, et al., 2013). Poor infant health outcomes may increase the likelihood of continued substance use/relapse among birthing people, thus increasing rates of family separation.	*“I can’t help but wonder if he’d still be the same giggly smiling kid if they would have taken him from me after birth.”—Person with lived experience of perinatal SUD*

**Table 3:** Feedback loops in the policy model

Name of feedback loop	Feedback loop structure	Explanation of feedback loop	Participant quotations
R9: Punitive policies encourage incarceration and structural stigma		Punitive policies refer to clinic, organizational, community or state policies that criminalize perinatal substance use, classify it as child abuse, or otherwise penalize the birthing person rather than provide necessary supportive services. A punitive policy environment increases the likelihood that a birthing person will be tested for substance use during or after pregnancy. Increased testing and punitive policies drive incarceration of birthing people for SUDs. As incarceration for substance use during pregnancy becomes more common, policymakers may use that as justification for enacting additional punitive policies.	*“Mandated toxicology testing for pregnant women and jailing women for substance use contributes to stigma and doesn’t fit with the paradigm of treating substance use disorder as a disease.” – OBGYN Provider*
R10: Basic needs must be met to benefit from treatment opportunities		Access to safe and supportive social, physical, and community environments encompasses all social determinants of health that are necessary for an individual to thrive, including access to education, employment, childcare, healthy and affordable food, housing, and other supports. Having these needs met increases a birthing person’s ability to access perinatal and SUD treatment, which lowers the likelihood of incarceration. Alternatively, incarceration takes away a birthing person’s access to a safe, supportive environment and limits their ability to connect to treatment and recovery supports, including medication for opioid use disorder.	*“We can’t emphasize enough the importance of comprehensive care, which includes access to healthy food, safe and affordable housing and a safe community in which to raise a family—without these things, it doesn’t matter how great our addiction and pregnancy care is, as we will not be able to meet all of the family’s vital needs.”—Maternal Fetal Medicine and Addiction Medicine Physician*

**Table 4:** Feedback loops in the workforce and evidence-based practices model

Name of feedback loop	Feedback loop structure	Explanation of feedback loop	Participant quotations
R11: Dissemination of evidence-based practices mitigate structural stigma		Awareness of evidence-based practices for perinatal SUD can grow through accessible training and dissemination efforts, which drives down structural stigma through increased institutional and organizational knowledge of SUD as a treatable medical condition. Lowering levels of structural stigma encourages the spread of evidence-based practices as systems are more likely to share and promote their knowledge and best practices.	*“At the end of the day, [the child welfare and criminal justice system] want the babies to be safe too…. But it’s how they’re trying to accomplish that that isn’t necessarily congruent with our evidence.”—Hospital Nursing Director of Women’s Health*
R12: Dissemination of evidence-based practices mitigate social stigma		Similarly to **R11**, a high level of awareness and accessibility of evidence-based practices works to change beliefs around perinatal SUD and reduces individual social stigma. As social stigma decreases, awareness and access to evidence-based practices increase.	*“When healthcare professionals have access to human stories, evidence-based policies and procedures, we start to see behaviors and attitudes change. We start to see healthcare professionals recognizing people with perinatal addiction as human. They start treating people with kindness again.”—Nurse-Midwife*
R13: Structural and social stigma can limit the influence of evidence-based practices		High levels of structural and social stigma can reduce the implementation of evidence-based practices. When providers and systems operationalize a false belief that SUDs represent an individual moral failing it thwarts the spread of evidence-based practices for perinatal SUD by reducing stigma-free training and knowledge sharing opportunities.	“*Our healthcare and criminal justice systems continue to operate under the idea that pregnant people should be able to stop using drugs when they get pregnant—even though an inability to stop using is a defining part of the disease of substance use disorders. Until we stop blaming people for their medical conditions at a system level, we will not allow pregnant people to consistently disclose their disease and access treatment.”—OBGYN Provider*

**Fig. 1:**
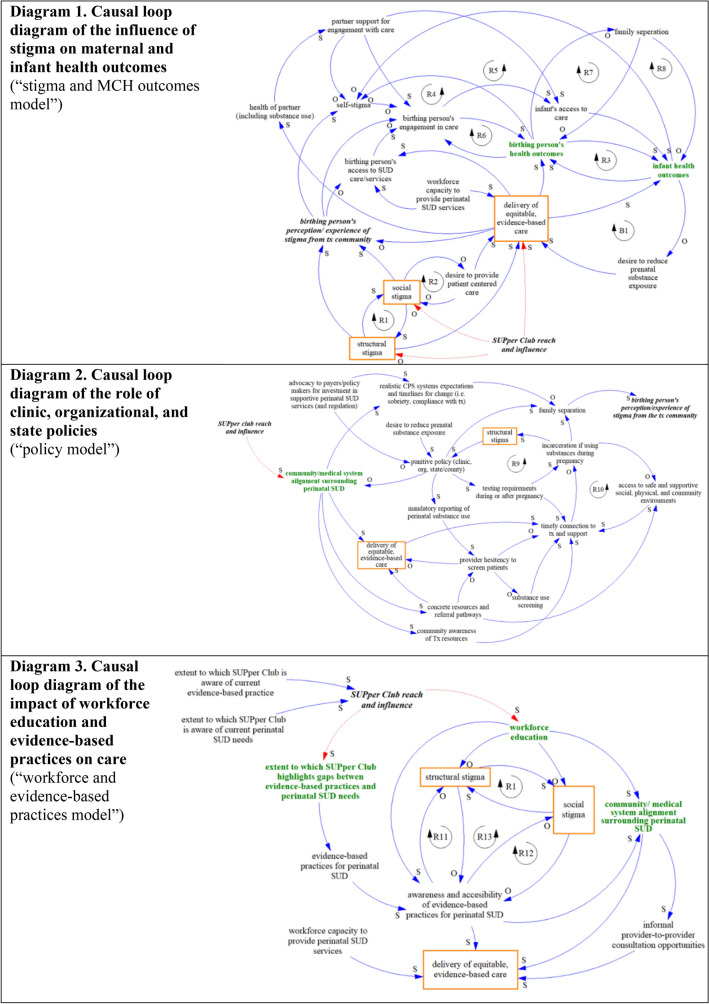
Causal loop diagrams of the Midwest SUPper Club’s role in the perinatal SUD system. *Bold, italicized text* was used to indicate the variables that were identified in BOTGs as the priority trends the group should focus on (“SUPper Club reach and influence”, “birthing person’s perceptions/experience of stigma from the tx community”). *Green* was used to indicate variables that the SUPper Club believes they can directly impact through the work of their coalition. *Orange* boxes were used to identify variables that the SUPper Club strives to indirectly impact through the work of their coalition. *Red dotted lines* indicate where participants determined the SUPper club should prioritize future efforts and potential pathways for the SUPper club’s work to most significantly influence the system. CLD’s identify relationships between variables using labeled arrows which indicate that a change in the first variable triggers a change in the second variable over time, all else equal. The polarity of causal links is labeled “S” to indicate the variables change in the same direction (e.g., if the value of the first goes up, the value of the second also goes up); they are labeled “O” to indicate that the variables change in opposite directions (e.g., if the value of the first goes up, the value of the second goes down) (Color figure online)

The first CLD (Fig. 1, with larger diagrams available in the Supplementary Material) illustrates the many ways in which stigma impacts maternal and infant health outcomes. Participants discussed various levels of stigma and the differing effect of internalized stigma versus stigmatization from a provider or a system-at-large. The diagram represents these dynamics in three variables: (1) *Self-stigma*: an acceptance of a negative narrative about the self; (2) *Social stigma*: individual stereotypes, prejudice, or discrimination (consciously or subconsciously), towards an already stigmatized group; and, (3) *Structural stigma*: policies and culture within institutions that promote negative attitudes towards a group, either directly or indirectly (Livingston et al., 2012; Trainor, 2022).

The second diagram (Fig. 1, with larger diagrams available in the Supplementary Material) resulted from rich discussions of the harmful impact of punitive policies and cross-system value and service alignment on perinatal SUD care. Timely connection to treatment and support emerged as a key outcome of interest. This requires both individuals’ basic needs (access to safe and supportive social, physical, and community environments) to be met as well as non-punitive evidence-based care (including screening/testing) to be provided. Connection to adequate treatment was found to be hindered by the fundamental system structure, which is not designed to engage or reach people who need perinatal SUD care. One health care provider shared an example of this: they reported hesitancy to screen patients for perinatal substance use due to inadequate referral options if use was identified. Additionally, a lack of awareness of treatment resources among perinatal people and their families, paired with fear of punitive consequences, leads to less timely connection to treatment and support.

The third diagram (Fig. 1, with larger diagrams available in the Supplementary Material) explores the impact of workforce education and evidence-based practices on the delivery of equitable, quality care. Workforce education in this context encompasses education on the impact of stigma, general knowledge on perinatal SUD, and information on available resources/referral sources and relevant laws and policies. Workforce education was emphasized for its potential to reduce stigma, increase systems alignment, and ultimately improve the quality of care delivered. The need for evidence-based practices for perinatal SUD was discussed broadly and in relation to caring for patients experiencing perinatal methamphetamine use. Unlike other types of substance use disorders such as opioid use disorder, methamphetamine poses significant challenges for treatment as it has few evidence-based treatment options (Courtney & Ray, 2014). It was also noted that the existence of evidence-based practices alone is not enough to influence the system—best practices must be made available through accessible training opportunities, supported at the systems- and organizational-level, and implemented alongside social and structural stigma reduction efforts.

**Fig. 2:**
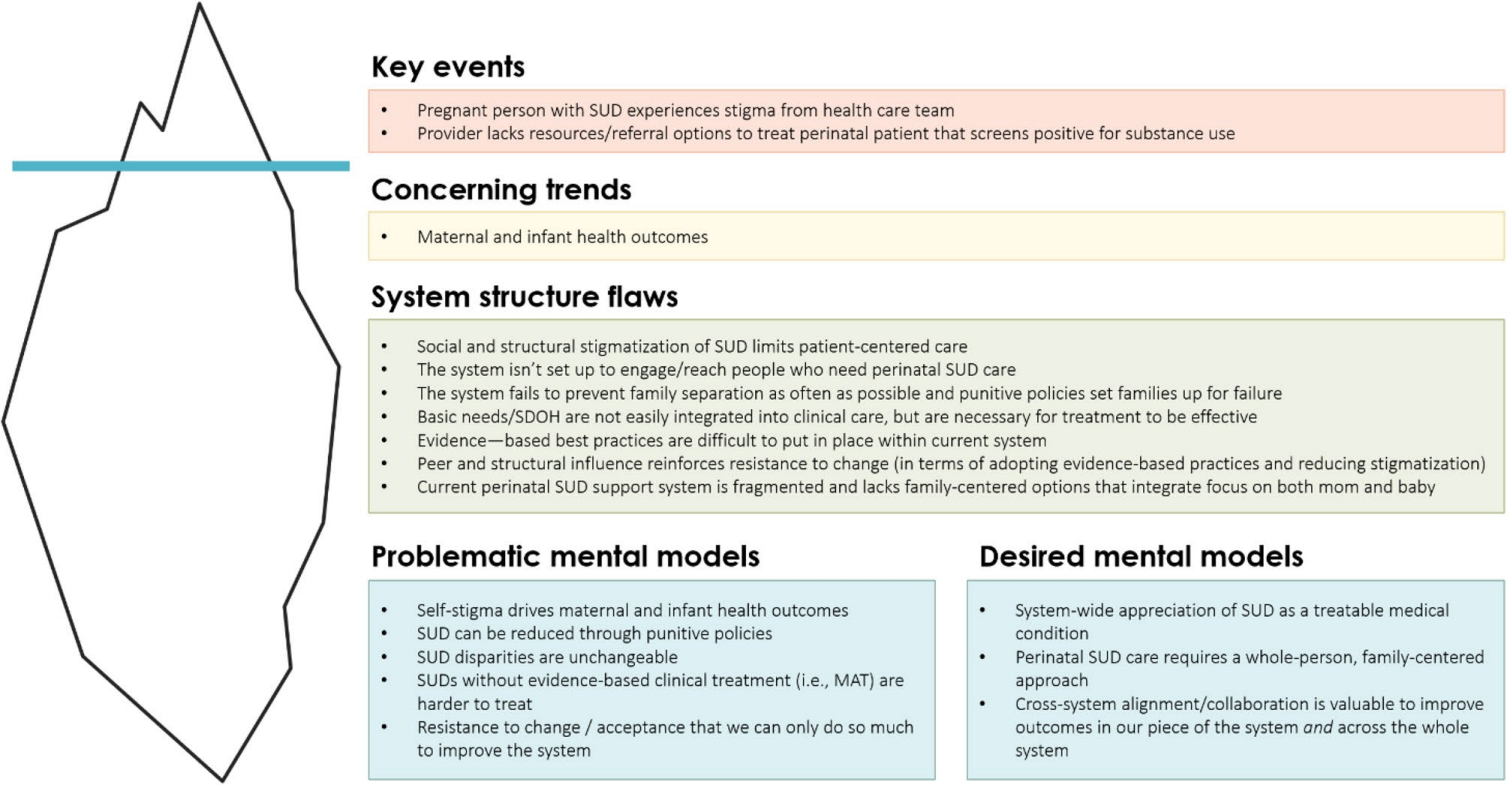
Synthesized GMB insights mapped to the iceberg framework. The Iceberg Framework is a common model used in systems thinking to represent the visible part of the problem above the water line, and the underlying trends, structure, and mental models under the water line (Maani & Cavana, 2007). This figure summarizes findings from the GMB workshop and interviews

After the workshop and interviews, the facilitation team developed a summary (Fig. 2) of system structure flaws and mental models (both problematic and desired) that emerged using the Iceberg Model of Systems Thinking (Maani & Cavana, 2007). The figure was validated by the SUPper club leadership and shared with all members. This summary is intended to support the SUPper Club in communicating and synthesizing key insights from the GMB process to stakeholders unfamiliar with CLDs.

### Leverage Points for Action

Based on the CLDs, participants developed and prioritized leverage points for action, or places within the system the SUPper Club can have the greatest impact. Four priority areas emerged from these conversations: (1) alignment and promotion of shared mental models across stakeholders, (2) expansion of workforce education and training opportunities for the perinatal SUD workforce, (3) strengthening of infrastructure to support care navigation for patients and providers, and (4) collaborative identification of evidence-based and regionally focused practices. Across all leverage points, there is a strong emphasis on centering patients and communities directly affected by perinatal SUD in all decision-making.

### Workshop Evaluation

Of the 20 participants who attended the workshop, 11 completed the post-workshop evaluation. 75% of respondents reported that participation in the workshop increased collaboration across the Midwest SUPper Club “to a high degree.” All respondents (N = 11) reported that participation in the workshop supported to a “high degree” or “very high degree” the development of a shared mental model of the most pertinent challenges facing perinatal people with SUD in the Midwest. One participant noted that the workshop “…helped [the Midwest SUPper Club] navigate what feels like an overwhelming amount of areas for us to work on, and instead focus on what people think is important for our organization.” Another participant reflected that the workshop “…really made me think about all the details of the work we do, the families and communities it affects, and the groundwork we need to accomplish before moving towards external funding support. This is definitely the motivation I needed to take the next step in our development.”

## Discussion

### The Influence of Stigma on Maternal and Infant Health Outcomes

The workshop and interviews highlighted ways in which stigmatization harms perinatal people with SUD and their infants and families, including through decreased access to services, care engagement, and effects on the overall quality of care provided (Patrick et al., 2017; Stone, 2015). One participant called attention to a structural flaw in the system that encourages providers and stakeholders to prioritize either maternal ***or*** infant needs, even though there is a broad recognition that maternal ***and*** infant health outcomes are deeply interrelated (Lander et al., 2013; Patrick et al., 2017). This points to the need for equitable, family-centered, non-judgmental perinatal SUD care that is well-coordinated and aligned across health and social services. Through the GMB process, the SUPper club determined that one of their key roles as a collaborative is to decrease stigma through workforce trainings, community education, and system-alignment efforts.

### The Role of Clinic, Organizational, and State Policies

Punitive policies create and uphold barriers to engagement in perinatal and SUD care. The term “punitive policies” was used by participants in the workshop to refer to both state-level policies (i.e., legislation classifying perinatal substance use as child abuse or neglect), and clinic/organizational policies (i.e., a mandate to report perinatal substance use to the criminal justice and/or child welfare system) that punish and/or criminalize perinatal substance use. Such policies deter pregnant or parenting people from seeking care and do not improve maternal or infant outcomes (Angelotta et al., 2016; Faherty et al., 2019; Patrick et al., 2017). Criminalization of perinatal SUD operationalizes the belief that substance use can be reduced through punitive policies, which reinforces a harmful and inequitable system structure. These policies set families up for failure (often creating vicious cycles—reinforcing feedback loops) and encourage family separation, particularly among low-income families of color. Participants discussed many ways in which shifting to a supportive system that promotes family unification can improve outcomes, including by reducing adverse childhood experiences and by increasing the likelihood of engagement in SUD treatment (Harp & Oser, 2018; Lander et al., 2013; Lollar, 2017).

Punitive policies also make it difficult for community systems (i.e., child protective services and housing providers) and medical systems (i.e., clinical providers and recovery programs) to align to support people affected by SUD during pregnancy. An example of this from the GMB workshop came from a public health professional who shared that often unrealistic expectations and requirements from child protective service agencies (i.e., mandating sobriety within a specific number of weeks) leads to family separation, temporarily or permanently. Identifying and eliminating these types of punitive policies was discussed as a major opportunity to stimulate meaningful cross-system alignment and collaboration.

### The Impact of Workforce Education and Evidence-Based Practices on Care

The GMB process illuminated the specific ways in which the SUPper Club seeks to strengthen the perinatal SUD system through cross-sector workforce education and the growth and dissemination of evidence-based practices. Participants discussed the overwhelming challenge of beginning to provide care for perinatal people with SUD, especially in organizations or systems that lack the necessary structure to adequately support implementation of perinatal SUD evidence-based treatment. The SUPper Club clarified their ideal role as both coordinators of regional workforce education opportunities and “boots on the ground,” meaning that the club aims to identify and call attention to gaps in perinatal SUD evidence-based practices and to highlight opportunities for and generate further research. In addition to formal evidence-based practices, informal provider-to-provider consultation emerged as an important factor in delivering equitable, evidence-based care.

### GMB as a Method to Accelerate Collaborative Planning Efforts

The GMB process, particularly CLD, is a powerful method to leverage diverse perspectives, equalize power dynamics and decision-making authority, visualize system structure, and collectively identify more influential leverage points for action. As the Midwest SUPper Club is in the early stages of development, GMB tools demonstrated a particular utility in building collaboration and reaching consensus across a broad range of stakeholder perspectives. For example, participants came in advocating for attention to issues nearest to their areas of expertise (e.g., “we too often neglect infants”), but left agreeing that stigma was a key fundamental issue to tackle first, and that conceptualizing their priorities as part of an inter-connected system strengthened all of their efforts. Further, these methods pushed participants to consider which perspectives were missing from the group. One participant reflected afterwards that “… since meeting with the MCH Workforce [Development Center], we were able to invite a member of the [local child protective services] team to attend our meetings, which broadens the perspective on this topic.” The SUPper Club plans to use the insights generated from this process to further grow and engage their membership and collaborate on best practice development, clinical outcomes research, and workforce learning opportunities (i.e., book clubs, annual conferences, and quarterly meetings).

This process has a few limitations. GMB workshops can be particularly challenging virtually and scheduling challenges led to non-homogenous participation across the 2 day workshop. Additionally, the virtual format required us to conduct our evaluation via a survey link rather than a paper survey; a limitation of our workshop was that only 55% of participants completed the evaluation survey. However, we feel we demonstrated scripted activities can still result in meaningful engagement in a virtual environment with a flexible participation structure. It should also be noted that although outreach was conducted to invite many individuals with lived experience to participate, ultimately only one person attended. This outreach was through personal contact and invitation of community-rooted organizations with whom individuals with lived experience had a trusted relationship.

GMB, specifically CLD, is reliant on skilled facilitators and takes practice to build competency. The GMB process is inherently iterative and should be enhanced with new perspectives and updated over time as the system changes. We support the on-going efforts in the system dynamics field to develop community-based GMB facilitators to inform and lead these efforts, and we believe that repeated exposure to systems thinking methodologies and utilization of the structured, scripted activities available can support the development of new facilitators (Hovmand, 2014; Hovmand et al., 2012, 2015). For this reason, the MCH Workforce Development Center prioritizes building systems thinking capacity and exposing MCH leaders, like those engaged in the SUPper Club, to the GMB process.

### Future Directions

This GMB process helped to illuminate the complexity of the perinatal SUD system in the largely rural Midwest and adds to the body of literature exploring substance use in rural America. Although the findings from this GMB process are specific to the Midwest, we believe the themes extracted are applicable to other regions. This demonstrates an opportunity to further explore and validate system dynamics in other perinatal SUD contexts using GMB to illicit perspectives and reach consensus on action steps to strengthen the system. We also see opportunities for future research and practice directed towards better understanding the system factors producing growing inequities in overdose death rates across sub-populations. Black and Indigenous people are disproportionately affected by fatal overdose, and a deeper understanding of the complexity of the issue and possible interventions is warranted (Kariisa et al., 2022). Lastly, while GMB evaluations, including those described in this paper, often focus on evaluation of workshop processes and outcomes (i.e., changes in mental models), future research should evaluate the long-term impact and sustainability of GMB-generated solutions on strengthening MCH and behavioral health systems (Felmingham et al., 2023).

As demonstrated in the present paper, the GMB process offers a pathway that could be employed broadly across the MCH system to bring together new partners and support a shared understanding of the problem and specific actions to improve outcomes of interest over time (Cilenti et al., 2019). We see great potential for the expanded use of this method among state and local MCH, clinical, and community-rooted partners to accelerate system-strengthening efforts through intentional collaboration and shared decision-making.

### Supplementary Information

Below is the link to the electronic supplementary material.

Supplementary file1 (DOCX 1074 kb)

## Data Availability

Not applicable.

## Footnotes

1 The Midwest SUPper Club defines the Midwest as the following states: Iowa, Illinois, Michigan, Minnesota, Missouri, Nebraska, North Dakota, Ohio, South Dakota, and Wisconsin. Current SUPper Club members represent Iowa, Michigan, Minnesota, South Dakota, and Wisconsin with the goal of engaging members from all 10 Midwestern states as the club continues to grow.

2 According to Ford (2019), Mental Models are “a relatively enduring and accessible, but limited, internal conceptual representation of a system (historical, existing, or projected) whose structure is analogous to the perceived structure of that system. Mental models represent the relationships and assumptions about a system held in a person's mind”.

3 The SUPper Club leadership is composed of a Certified Nurse Midwife, Social Worker, and OBGYN. All members of the SUPper Club leadership are authors on this paper.
