# Supplementary material for: Strengthening the System Supporting Perinatal People with Substance Use Disorder in the Midwest Using Group Model Building
Source: Matern Child Health J. 2023 Jul 21;27(Suppl 1):128–42. doi: 10.1007/s10995-023-03751-z (PMC10692270; doi:10.1007/s10995-023-03751-z)
Supplement: Supplementary file 1 — Supplementary file1 (DOCX 1074 kb) [file 10995_2023_3751_MOESM1_ESM.docx]

**Figure 1. Behavior Over Time Graphs**

Participants brainstormed and graphed the most important trends they would like to influence. Through a series of conversations, two primary trends (i.e., reference modes) were selected to focus on:

1. **The Midwest Supper Club’s reach and influence**

**
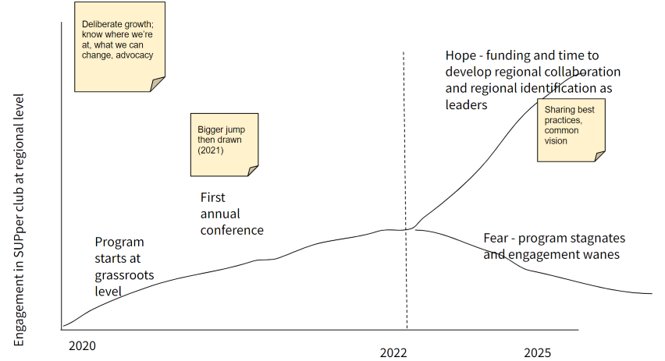
**

1.
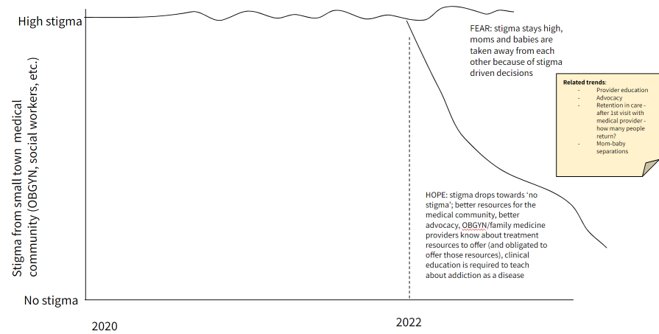
**Birthing person’s perception/experience of stigma from the treatment community**

**Figure 2. Causal loop diagram of the influence of stigma on maternal and infant health outcomes (“stigma and MCH outcomes model”)**


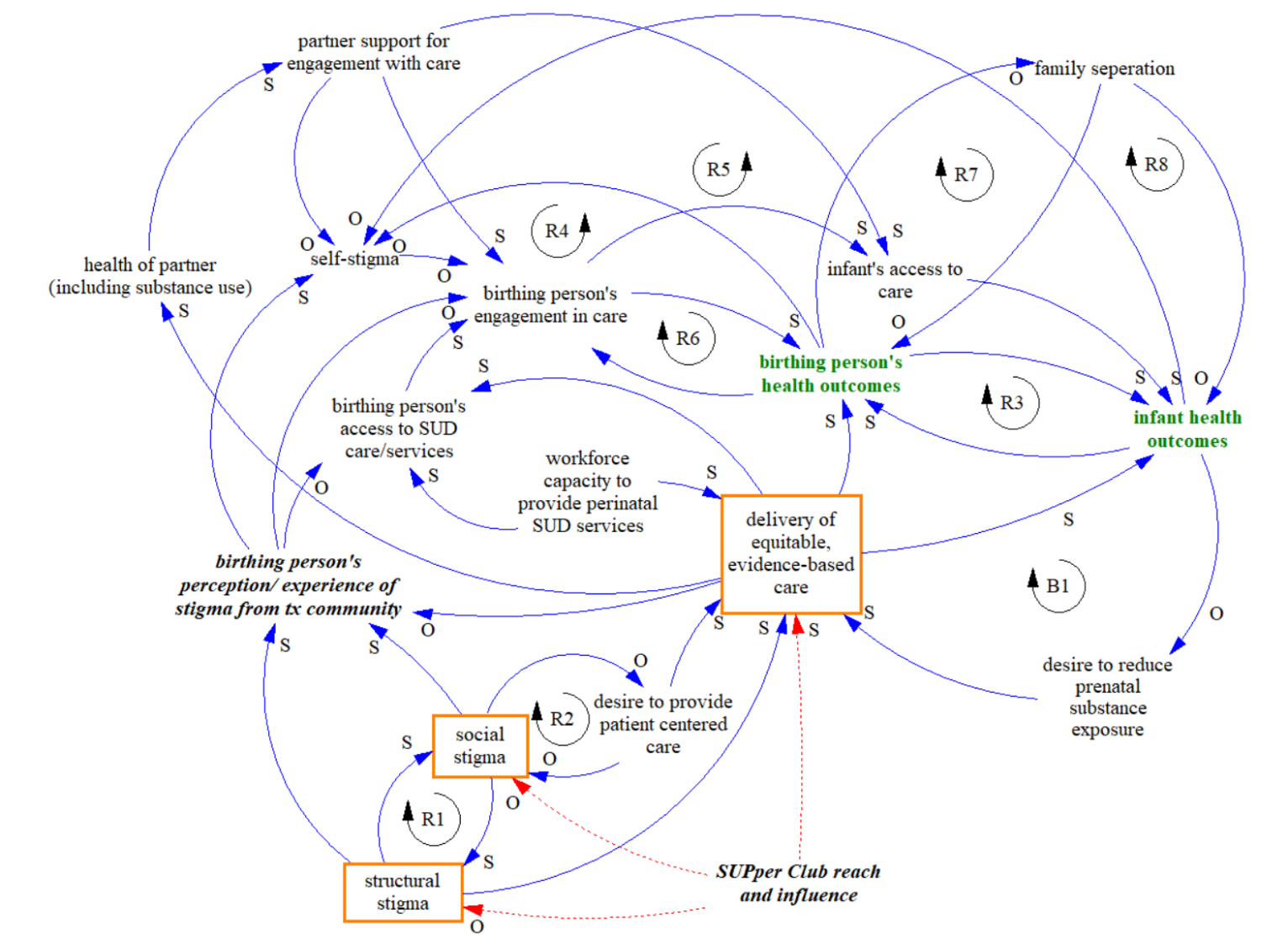


**Figure 2 caption**: ***Bold, italicized text*** was used to indicate the variables that were identified in BOTGs as the priority trends the group should focus on (“SUPper Club reach and influence”, “birthing person’s perceptions/experience of stigma from the tx community”). ***Green*** was used to indicate variables (“birthing person’s health outcomes”, “infant health outcomes”) that the SUPper Club believes they can directly impact through the work of their coalition. ***Orange*** boxes were used to identify variables (“structural stigma”, “social stigma”, “delivery of equitable, evidence-based care”) that the SUPper Club strives to indirectly impact through the work of their coalition. ***Red dotted lines*** indicate where participants determined the SUPper club should prioritize future efforts and potential pathways for the SUPper club’s work to most significantly influence the system. *Note: CLD’s identify relationships between variables using labeled arrows which indicate that a change in the first variable triggers a change in the second variable over time, all else equal. The polarity of causal links is labeled “S” to indicate the variables change in the same direction (e.g., if the value of the first goes up, the value of the second also goes up); they are labeled “O” to indicate that the variables change in opposite directions (e.g., if the value of the first goes up, the value of the second goes down).


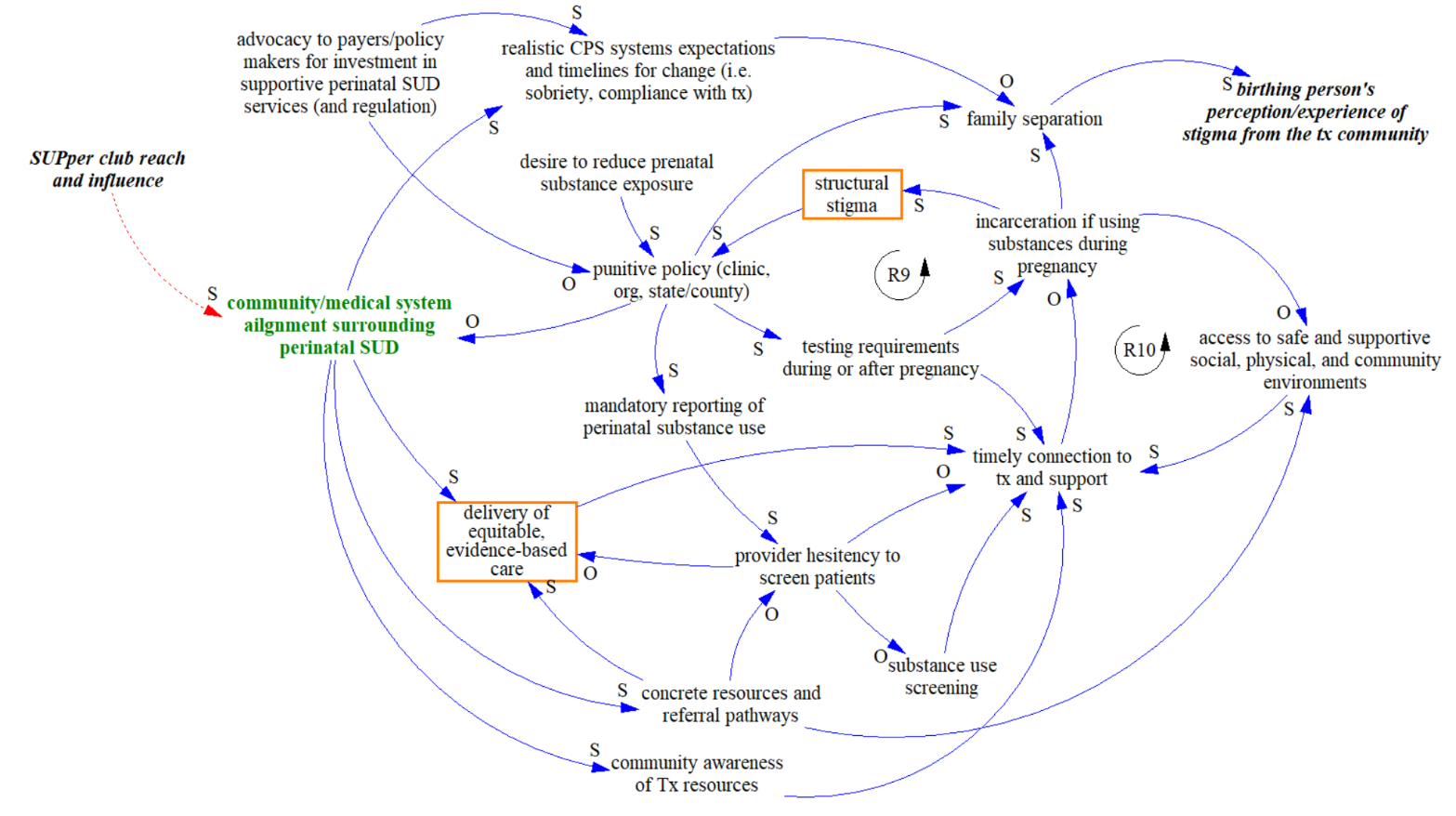
**Figure 3. Causal loop diagram of the role of clinic, organizational, and state policies (“policy model”)**

**Figure 3 caption**: ***Bold, italicized text*** was used to indicate the variables that were identified in BOTGs as the priority trends the group should focus on (“SUPper Club reach and influence”, “birthing person’s perceptions/experience of stigma from the tx community”). ***Green*** was used to indicate variables (“community/medical system alignment surrounding perinatal SUD”) that the SUPper Club believes they can directly impact through the work of their coalition. ***Orange*** boxes were used to identify variables (“structural stigma”, “delivery of equitable, evidence-based care”) that the SUPper Club strives to indirectly impact through the work of their coalition. ***Red dotted lines*** indicate where participants determined the SUPper club should prioritize future efforts and potential pathways for the SUPper club’s work to most significantly influence the system. *Note: CLD’s identify relationships between variables using labeled arrows which indicate that a change in the first variable triggers a change in the second variable over time, all else equal. The polarity of causal links is labeled “S” to indicate the variables change in the same direction (e.g., if the value of the first goes up, the value of the second also goes up); they are labeled “O” to indicate that the variables change in opposite directions (e.g., if the value of the first goes up, the value of the second goes down).

**Figure 4.** **Causal loop diagram of the impact of workforce education and evidence-based practices on care (“workforce and evidence-based practices model”).**


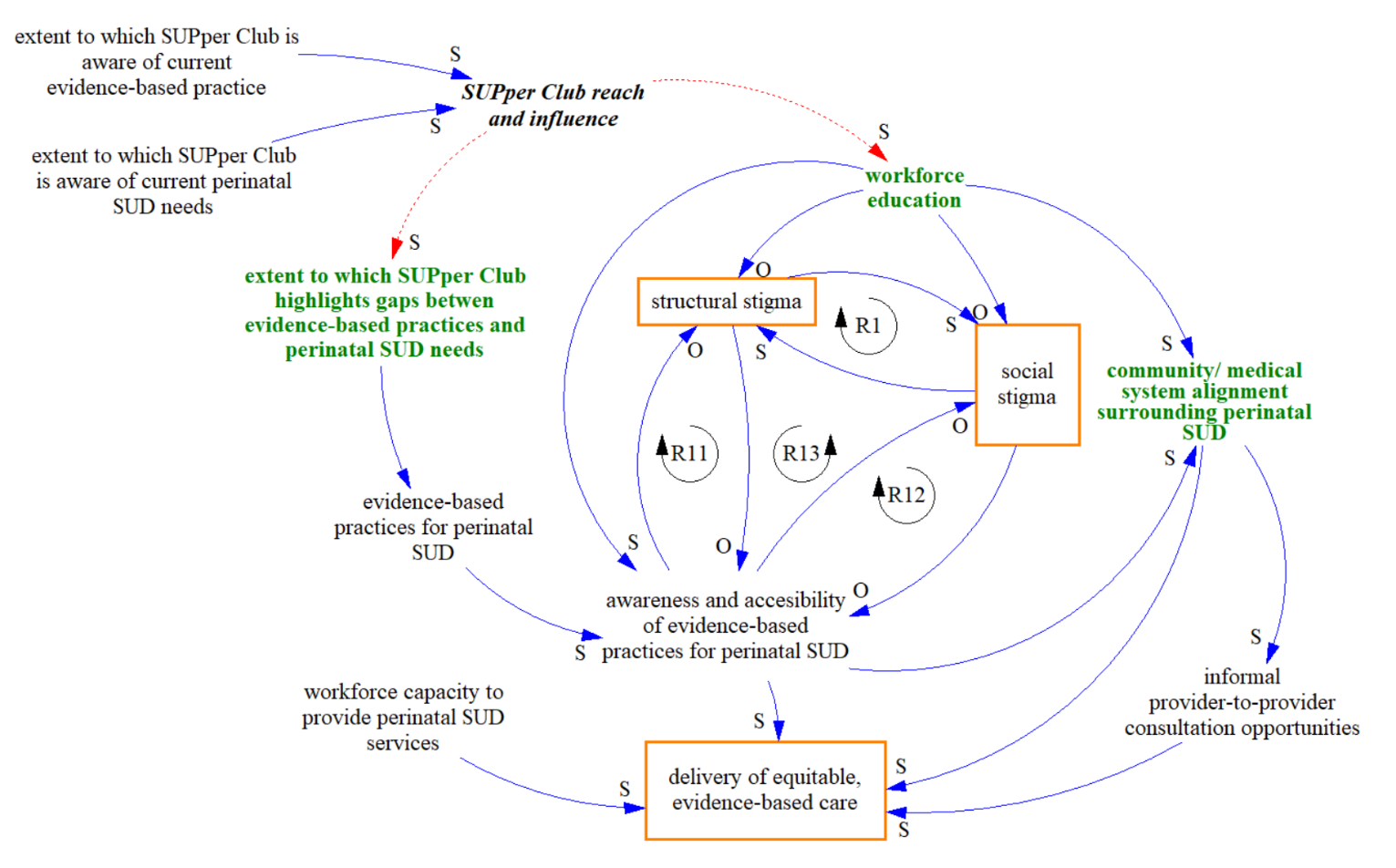


**Figure 4 caption**: ***Bold, italicized text*** was used to indicate the variables that were identified in BOTGs as the priority trends the group should focus on (“SUPper Club reach and influence”). ***Green*** was used to indicate variables (“community/medical system alignment surrounding perinatal SUD”, “workforce education”, and “extent to which SUPper Club highlights gaps between evidence-based practices and perinatal SUD needs”) that the SUPper Club believes they can directly impact through the work of their coalition. ***Orange*** boxes were used to identify variables (“structural stigma”, “social stigma”, and “delivery of equitable, evidence-based care”) that the SUPper Club strives to indirectly impact through the work of their coalition. ***Red dotted lines*** indicate where participants determined the SUPper club should prioritize future efforts and potential pathways for the SUPper club’s work to most significantly influence the system. *Note: CLD’s identify relationships between variables using labeled arrows which indicate that a change in the first variable triggers a change in the second variable over time, all else equal. The polarity of causal links is labeled “S” to indicate the variables change in the same direction (e.g., if the value of the first goes up, the value of the second also goes up); they are labeled “O” to indicate that the variables change in opposite directions (e.g., if the value of the first goes up, the value of the second goes down).

**Table 1. Variable List: Stigma and MCH Outcomes Model**

| **Variable** | **Type** |
| --- | --- |
| SUPper Club reach and influence | Exogenous* |
| Partner support for engagement with care | Endogenous** |
| Self-stigma | Endogenous** |
| Health of partner (including substance use) | Endogenous** |
| Birthing person’s access to SUD care/services | Endogenous** |
| Birthing person’s engagement in care | Endogenous** |
| Desire to provide patient centered care | Endogenous** |
| Birthing person’s health outcomes | Endogenous** |
| Baby’s access to care | Endogenous** |
| Infant health outcomes | Endogenous** |
| Workforce capacity to provide perinatal SUD services | Endogenous** |
| Social stigma | Endogenous** |
| Family separation | Endogenous** |
| Birthing person’s perception/experience of stigma from tx community | Endogenous** |
| Desire to reduce prenatal substance exposure | Endogenous** |
| Delivery of equitable, evidence-based care | Endogenous** |
| Structural stigma | Endogenous** |

***Exogenous variable/view:** *external, the opposite of endogenous. An exogenous view assumes that a system's behavior is dominated by the influence of outside forces or factors. An exogenous variable is an external (input) variable that affects but is not affected by the system.^1^*

****Endogenous variable/view:** *internal, the opposite of exogenous. An endogenous view approaches a problem searching for its causes and solutions within the system boundary. Endogenous variables are affected by other system variables.^1^*

Citation:

1. Ford, D.N. (2019), A system dynamics glossary. Syst. Dyn. Rev., 35: 369-379. <https://doi.org/10.1002/sdr.1641>

**Table 2. Variable List: Policy Model**

| **Variable** | **Type** |
| --- | --- |
| SUPper Club reach and influence | Exogenous* |
| Advocacy to payers/policy makers for investment in supportive perinatal SUD services (and regulation) | Exogenous* |
| Family separation | Endogenous** |
| Birthing person’s perception/experience of stigma from tx community | Endogenous** |
| Desire to reduce prenatal substance exposure | Endogenous** |
| Delivery of equitable, evidence-based care | Endogenous** |
| Structural stigma | Endogenous** |
| Community/medical system alignment surrounding perinatal SUD | Endogenous** |
| Realistic CPS systems expectations and timelines for change (i.e. sobriety, compliance with tx) | Endogenous** |
| Punitive policy (clinic, org, state/county) | Endogenous** |
| Mandatory reporting of perinatal substance use | Endogenous** |
| Provider hesitancy to screen patients | Endogenous** |
| Concrete resources and referral pathways | Endogenous** |
| Community awareness of Tx resources | Endogenous** |
| Substance use screening | Endogenous** |
| Timely connection to tx and support | Endogenous** |
| Testing requirements during or after pregnancy | Endogenous** |
| Incarceration if using substances during pregnancy | Endogenous** |
| Access to safe and supportive social, physical, and community environments | Endogenous** |

***Exogenous variable/view:** *external, the opposite of endogenous. An exogenous view assumes that a system's behavior is dominated by the influence of outside forces or factors. An exogenous variable is an external (input) variable that affects but is not affected by the system.^1^*

****Endogenous variable/view:** *internal, the opposite of exogenous. An endogenous view approaches a problem searching for its causes and solutions within the system boundary. Endogenous variables are affected by other system variables.^1^*

Citation:

1. Ford, D.N. (2019), A system dynamics glossary. Syst. Dyn. Rev., 35: 369-379. <https://doi.org/10.1002/sdr.1641>

**Table 3. Variable List: Workforce and Evidence-Based Practices Model**

| **Variable** | **Type** |
| --- | --- |
| Workforce capacity to provide perinatal SUD services | Exogenous* |
| Extent to which SUPper Club is aware of current evidence-based practice | Exogenous* |
| Extent to which SUPper Club is aware of current perinatal SUD needs | Exogenous* |
| Social stigma | Endogenous** |
| Delivery of equitable, evidence-based care | Endogenous** |
| Structural stigma | Endogenous** |
| SUPper Club reach and influence | Endogenous** |
| Community/medical system alignment surrounding perinatal SUD | Endogenous** |
| Workforce education | Endogenous** |
| Extent to which SUPper Club highlights gaps between evidence-based practices and perinatal SUD needs | Endogenous** |
| Evidence-based practices for perinatal SUD | Endogenous** |
| Awareness and accessibility of evidence-based practices for perinatal SUD | Endogenous** |
| Informal provider-to-provider consultation opportunities | Endogenous** |

***Exogenous variable/view:** *external, the opposite of endogenous. An exogenous view assumes that a system's behavior is dominated by the influence of outside forces or factors. An exogenous variable is an external (input) variable that affects but is not affected by the system.^1^*

****Endogenous variable/view:** *internal, the opposite of exogenous. An endogenous view approaches a problem searching for its causes and solutions within the system boundary. Endogenous variables are affected by other system variables.^1^*

Citation:

1. Ford, D.N. (2019), A system dynamics glossary. Syst. Dyn. Rev., 35: 369-379. <https://doi.org/10.1002/sdr.1641>
